# Prevalence, Risk Factors and Potential Protective Strategies for Hypomagnesemia in Kidney Transplant Recipients

**DOI:** 10.3390/ijms26136528

**Published:** 2025-07-07

**Authors:** Cristina Riaza Ortiz, Carlos Fernández Fernández, Marina Pujol Pujol, María Muñiz Rincón, Arianne Sofía Aiffil Meneses, Isabel María Pérez Flores, Natividad Calvo Romero, María Ángeles Moreno de la Higuera, Beatriz Rodríguez Cubillo, Raquel Ramos Corral, Ana Isabel Sánchez Fructuoso

**Affiliations:** 1 Nephrology Department, San Carlos Clinical University Hospital, 28040 Madrid, Spain; carlosffernande@salud.madrid.org (C.F.F.); marina.pujol@salud.madrid.org (M.P.P.); maria.muniz@salud.madrid.org (M.M.R.); ariannesofia.aiffil@salud.madrid.org (A.S.A.M.); isabelmaria.perez@salud.madrid.org (I.M.P.F.); natividad.calvo@salud.madrid.org (N.C.R.); m.angeles.morenodelahiguera@salud.madrid.org (M.Á.M.d.l.H.); brodriguezc@salud.madrid.org (B.R.C.); asfructuoso@salud.madrid.org (A.I.S.F.); 2 Biomedical Research Institute of San Carlos Clinical University Hospital, 28040 Madrid, Spain; rrcorral@salud.madrid.org; 3 Department of Medicine, Complutense University, 28040 Madrid, Spain; 4 Clinical Analysis Department, San Carlos Clinical University Hospital, 28040 Madrid, Spain

**Keywords:** magnesium, kidney transplant, tacrolimus, thiazides, cinacalcet, SGLT2i

## Abstract

Hypomagnesemia is the most common electrolyte disorder in kidney transplant recipients (KTR), yet its causes remain unclear. Few studies have explored its underlying factors. This study aimed to assess its prevalence and identify risk factors in KTR. We conducted a retrospective cross-sectional study in 489 outpatient KTR. Demographic, clinical, and laboratory data were collected. Univariate and multivariate logistic regression analyses were used to identify factors associated with hypomagnesemia (≤1.7 mg/dL). Hypomagnesemia was present in 50.7% of patients. Multivariate analysis identified tacrolimus [OR 2.91 (1.62–5.22)], thiazides [OR 2.23 (1.21–4.08)], cinacalcet [OR 2.31 (1.29–4.13)], serum phosphate < 3.7 mg/dL [1.99 (1.29–3.05)], serum calcium ≤ 10 mg/dL [1.99 (1.29–3.05)] and diabetes [1.94 (1.22–3.08)] as risk factors. Protective factors included SGLT2 inhibitors (SGLT2i) [OR 0.17 (0.10–0.27)] and mTOR inhibitors (mTORi) [OR 0.62 (0.38–0.98)]. Among hypomagnesemic patients, those receiving Mg^2+^ supplements had lower Mg^2+^ levels [1.54 (0.15) vs. 1.59 (0.13) mg/dL, *p* = 0.005] and higher fractional Mg^2+^ excretion [8.28 (4.48)% vs. 7.36 (4.19)%, *p* = 0.05]. Hypomagnesemia is highly prevalent in KTR. Tacrolimus, thiazides, and cinacalcet are key risk factors and, in some patients, risks and benefits of continuing these medications should be carefully weighed. In refractory cases, SGLT2i or mTORi may offer benefit.

## 1. Introduction

Kidney transplantation is the most effective treatment for end-stage kidney disease. In recent decades, graft and patient survival have improved despite challenges such as older recipient age, higher body mass index, diabetes mellitus (DM) prevalence, and HLA presensitization. Additionally, donor characteristics have worsened, with increased donor age and more circulatory death donations [1]. Kidney transplantation is associated with many complications, with immunosuppression management being perhaps the most important due to its link with rejection, infections, and an increased risk of cardiovascular disease. Proper management of electrolyte disorders is also crucial, as they are frequently reported in this population, with hypomagnesemia being the most common [2,3,4,5]. Despite its high frequency, the literature about this topic remains scarce.

Magnesium (Mg^2+^) is the fourth most abundant cation in the body and the second most abundant at the intracellular level, playing a role in numerous cellular physiological processes [6]. Hypomagnesemia has been linked to cardiovascular disease (CVD) through various mechanisms, including hyperglycemia, chronic inflammation, hypertension and vascular dysfunction.

Mg^2+^ levels depend on intestinal absorption, bone deposition, and renal excretion. Under normal conditions, 30–50% [7,8] of Mg^2+^ is absorbed in the intestine.

In the kidneys, 2400 mg of Mg^2+^ is filtered daily, with 95–99% reabsorbed [8]. Numerous drugs have been associated with hypomagnesemia [7,9,10], including insulin and epinephrine (which promote intracellular Mg^2+^ uptake), laxatives, proton pump inhibitors (PPIs), and metformin (through gastrointestinal losses), as well as antibiotics, antifungals, and antivirals; antineoplastic agents like carboplatin and cisplatin; calcineurin inhibitors; thiazides; furosemide; and digoxin (which increase urinary excretion) [8].

Diagnosing hypomagnesemia can be challenging, as clinical manifestations are often nonspecific and, in many cases, asymptomatic. As nephrologists, it is our responsibility to diagnose it, as it is the most common electrolyte disorder. Symptoms may include weakness, confusion, neuromuscular irritability, vertical nystagmus, athetosis, and depression. In severe cases (<0.5 mmol/L or 1.2 mg/dL), symptoms may be more serious, including tetany, seizures, psychosis, and arrhythmias (atrial fibrillation, supraventricular tachycardia, torsades de pointes).

Different review articles [11,12,13] suggest that hypomagnesemia is associated with critical health issues, including but not limited to hypertension, CVD, type 2 DM, and osteoporosis. A retrospective cohort study in heart transplant (HT) recipients receiving calcineurin inhibitors showed that 15 years post-transplant, both survival and freedom from cardiac allograft vasculopathy (CAV) were worse in patients with hypomagnesemia. Multivariate analyses consistently demonstrated that low serum Mg^2+^ was independently associated with a 2.6-fold increased risk of mortality and a 4-fold increased risk of CAV (95% CI 1.06 to 6.4, *p* = 0.04; 95% CI 1.12 to 14.42, *p* = 0.01, respectively). In conclusion, low serum Mg^2+^ is independently associated with increased mortality and CAV in HT patients [14].

Given the clinical relevance of hypomagnesemia in KTR and the limited evidence regarding its underlying causes, we conducted a retrospective, cross-sectional study based on the hypothesis that certain immunosuppressive agents and comorbidities might be independently associated with lower serum Mg^2+^ levels.

The aim of our study was to determine the prevalence of hypomagnesemia in a stable outpatient KTR cohort and identify potential risk factors for hypomagnesemia. In a second phase we explored the potential impact of Mg^2+^ supplementation and we aimed to clarify the profile of patients at highest risk and to support future clinical strategies for monitoring and management.

## 2. Results

We evaluated 489 patients. Hypomagnesemia was the most frequent electrolyte disturbance (50.7%), followed by metabolic acidosis (13.2%) and hyperkalemia (5.7%).

The median serum magnesium (sMg^2+^) level was 1.70 mg/dL (IQR: 1.60–1.90). In accordance with the ranges of our laboratory and the median value of all sMg^2+^ levels, patients were divided into normal sMg^2+^ (>1.7 mg/dL, N = 241) and low (≤1.7 mg/dL, N = 248) sMg^2+^ groups. Patients with normal sMg^2+^ had a mean sMg^2+^ of 1.96 (0.17) mg/dL, while patients with low sMg^2+^ had a mean of 1.56 (0.14) mg/dL (*p* < 0.001). There were no differences between age, gender, prevalence of DM, time post-transplantation or eGFR. Table 1 and Table 2 summarize the characteristics of the patients.

**Figure 1:**
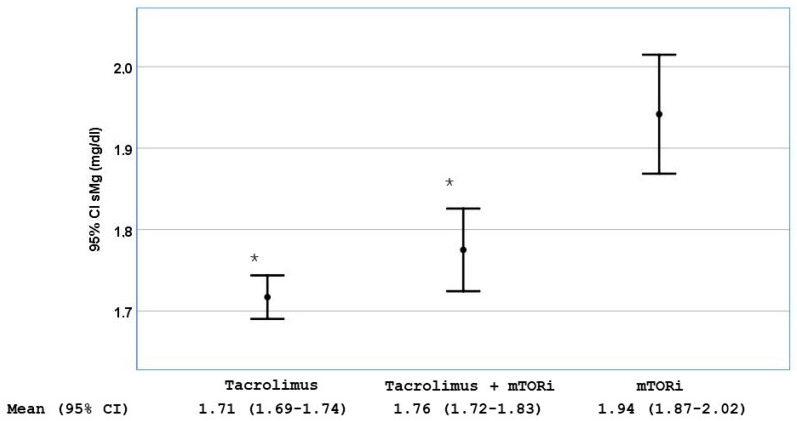
Serum magnesium (sMg^2+^) levels stratified by the use of tacrolimus alone, MTORi alone or the combination of both. Data are shown as mean with 95% CI; * *p* < 0.001 compared with the group of patients treated only with MTORi.

We found that patients treated with tacrolimus had lower mean sMg^2+^ levels than those treated with mTORi [1.71 mg/dL (95% CI 1.69–1.74) vs. 1.94 mg/dL (95% CI 1.87–2.02), *p* < 0.001]. The combination of mTORi with tacrolimus did not result in a statistically significant increase in sMg^2+^ levels [1.76 mg/dL (95% CI 1.72–1.83), *p* = 0.151 vs. tacrolimus alone] (Figure 1). Tacrolimus levels were higher in patients with hypomagnesemia [8.22 ng/dL (95% CI 7.85–8.56) vs. 7.68 mg/dL (95% CI 7.39–7.89), *p* = 0.03].

**Figure 2:**
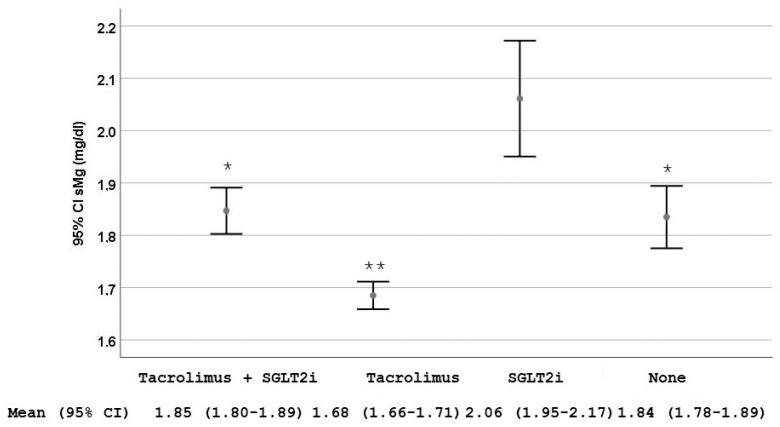
Serum magnesium (sMg^2+^) levels stratified by the use of tacrolimus alone, sodium-glucose cotransporter-2 inhibitors (SGLT2i) alone, the combination of both and none of them. Data are shown as mean and 95% CI. * *p* = 0.002 compared with the group treated only with SGLT2i; ** *p* < 0.001 compared with the other three groups.

We observed that patients treated with tacrolimus and sodium-glucose cotransporter-2 inhibitors (SGLT2i) had sMg^2+^ levels higher than those on tacrolimus without SGLT2i [1.85 mg/dL (95% CI 1.80–1.89) vs. 1.68 mg/dL (95% CI 1.66–1.71), *p* < 0.001] (Figure 2).

**Figure 3:**
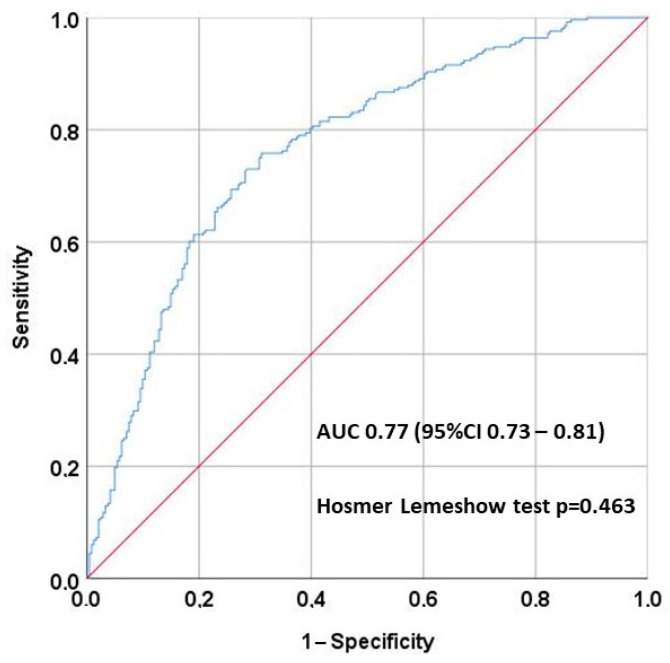
The area under the multivariate analysis curve was 0.77 (95% CI 0.73–0.81), showing good discrimination, and the Hosmer–Lemeshow test *p* = 0.463, showing good calibration.

Logistic regression analysis (Table 3) showed risk factors for hypomagnesemia. The risk was higher in patients receiving tacrolimus [OR 2.91 (95% CI 1.62–5.22)], thiazides [OR 2.23 (95% CI 1.21–4.08)], and cinacalcet [OR 2.31 (95% CI 1.29–4.13)], whereas the use of SGLT2i and mTORi had a protective effect [OR 0.17 (0.10–0.27) and 0.62 (0.38–0.98, respectively]. The area under the multivariate analysis curve was 0.77 (95% CI 0.73–0.81) (Figure 3), and the Hosmer–Lemeshow test *p* = 0.463.

In our study population, there were 249 patients with DM, of whom 98 (39.4%) were treated with SGLT2i. The prevalence of hypomagnesemia was 63.6% in those not receiving SGLT2i compared to 27.6% in those treated, with mean sMg^2+^ levels of 1.69 (0.24) mg/dL vs. 1.88 (0.25) mg/dL, respectively (*p* < 0.001). Regarding renal function, the mean eGFR by CKD-EPI was 45.3 (20.8) mL/min/1.73 m^2^ in patients not receiving SGLT2i and 48.1 (21.1) mL/min/1.73 m^2^ in those treated, with no statistically significant difference (*p* = 0.304). Hypomagnesemia prevalence by eGFR category was 48.3% in patients with eGFR < 32 mL/min/1.73 m^2^, 49.6% in those with 32–45 mL/min/1.73 m^2^, 46.6% in the 45–61 mL/min/1.73 m^2^ group, and 58.1% in those with eGFR > 61 mL/min/1.73 m^2^, with no statistically significant differences among groups (*p* = 0.282)

Patients treated with oral Mg^2+^ supplements had lower sMg^2+^ levels than those not supplemented [1.62 (0.21) vs. 1.81 (0.24) mg/dL, *p* < 0.001], with a higher fractional excretion of Mg^2+^ [8.24 (4.2) % vs. 7.37 (3.5) %, *p* = 0.038] compared to those who did not receive oral supplements. Among hypomagnesemic patients, those receiving oral Mg^2+^ supplementation had slightly lower Mg^2+^ levels [1.54 (0.15) vs. 1.59 (0.13) mg/dL, *p* = 0.005] and higher fractional Mg^2+^ excretion [8.28 (4.48) % vs. 7.36 (4.19) %, *p* = 0.05].

## 3. Discussion

Our study confirms that hypomagnesemia is the most frequent hydroelectrolytic disturbance in kidney transplant recipients (KTR) [2,3,5,15,16].

We have confirmed previous studies showing the negative effect of tacrolimus on sMg^2+^. As mentioned before, Mg^2+^ is filtered in the kidneys, with 95–99% reabsorbed [8]: 20–30% in the proximal tubule, mainly through passive paracellular transport, 65% in the ascending loop of Henle through voltage gradient-dependent paracellular passive transport, and the remaining 5–10% in the distal convoluted tubule (DCT) via active transcellular transport facilitated by the Transient Receptor Potential Melastatin 6 (TRPM6) channel. It is known that tacrolimus promotes downregulation of the TRPM6, and it has been shown that tacrolimus decreases Mg^2+^ levels to a greater extent compared to cyclosporine [5,17]. In our cohort, patients with hypomagnesemia had significantly higher tacrolimus levels compared to those with normal serum Mg^2+^ (8.22 vs. 7.68 ng/mL, *p* = 0.03), suggesting a potential dose-dependent effect.

In contrast with other studies, we did not find an association between hypomagnesemia and PPIs in our patients, as previously described (Table 2). This may be due to a high percentage of patients receiving these drugs as well as other factors such as tacrolimus, SGLT2i, and thiazides which may have masked their effect. Although we cannot confirm whether patients actually took the PPIs, we verified that the medication was collected from the pharmacy. One of the causes of hypomagnesemia we identified is the use of thiazides (Table 3). This diuretic class, widely used for volume and blood pressure control, acts on the NaCl cotransporter in the distal convoluted tubule. Its adverse effects include hypokalemia, hyponatremia, and hypomagnesemia [18]. Thus, it may be reasonable to consider alternative diuretics in our patients, particularly those on tacrolimus-based immunosuppression.

The use of cinacalcet was associated with decreased serum Mg^2+^ levels in our KTR. Many years ago, Zitt et al. [19] demonstrated an increase in urinary sodium, calcium, and Mg^2+^ concentration after cinacalcet administration in kidney transplant patients with persistent hyperparathyroidism. Additionally, lower phosphorus levels below our 75th percentile (<3.7 mg/dL) were associated with hypomagnesemia, likely reflecting underlying hyperparathyroidism.

Some authors have described that mTORi can cause hypomagnesemia via renal Mg^2+^ wasting, although these are experimental studies on acute drug toxicity [20]. However, in a retrospective study including 138 renal transplant patients converted from CNIs to mTORi over six months, our group previously described that serum Mg^2+^ concentration significantly increased along with a reduction in the fractional excretion of Mg^2+^ [21]. The present study confirms that hypomagnesemia is more frequent in patients treated with tacrolimus than in those treated with mTORi (Figure 1 and Table 2).

Conversely, we found a beneficial effect of SGLT2i on hypomagnesemia, as previously described [22,23,24]. Different mechanisms have been proposed to explain this association (reviewed in [25]). First, increased Na^+^ delivery to the thick ascending limb of the loop of Henle can raise transmembrane potential gradient from increased activity of the Na^+^-K^+^ 2Cl^−^ cotransporter, driving passive paracellular Mg^2+^ reabsorption. Second, increased tubular flow and changes in electrolyte handling may stimulate Mg^2+^ transport in the distal nephron. Third, increased epidermal growth factor (EGF), glucagon, sodium/chloride cotransporter activation, and uromodulin, along with decreased insulin resistance and reactive oxygen species, can directly enhance TRPM6-mediated transepithelial Mg^2+^ transport in DCT cells. Reduced circulating insulin levels may shift Mg^2+^ from the intracellular to the extracellular compartment. Systemic factors influenced by SGLT2i (elevated EGF, glucagon, and uromodulin, and reduced insulin resistance and reactive oxygen species (ROS)) may also enhance Mg^2+^ absorption via TRPM6 and Transient Receptor Potential cation channel subfamily M member 7-mediated active transport in the gut [25]. The effect of these drugs has been demonstrated in KTR on tacrolimus [26] and in patients with refractory hypomagnesemia [25,27]. Evidence from animal models also supports the beneficial effect of SGLT2i on magnesium homeostasis. In a study using rats with metabolic syndrome induced by a high-fructose diet, treatment with dapagliflozin significantly increased serum Mg^2+^ levels compared to untreated controls [25]. These findings corroborate clinical observations and reinforce the hypothesis of a renal and systemic mechanism of action [25].

Regarding the magnesium handling between diabetic and non-diabetic patients, our subgroup analysis showed that diabetic patients treated with SGLT2i had higher medium sMg^2+^ levels (1.88 vs. 1.69 mg/dL) and a 36% lower prevalence of hypomagnesemia compared to those not receiving these agents (*p* < 0.001 for both comparisons). In contrast, we did not observe statistically significant differences in hypomagnesemia prevalence across categories of renal function. Although impaired renal function could theoretically reduce magnesium excretion, our data suggest that in this population, eGFR alone does not appear to be a strong determinant of magnesium levels. This suggests that magnesium balance in KTR may be influenced by a complex interplay of factors such as immunosuppressive therapy, gastrointestinal losses or individual variability in tubular handling of magnesium.

It is widely accepted that Mg^2+^ supplements should be prescribed to hypomagnesemic patients; however, their absorption and bioavailability are low, and gastrointestinal intolerance often renders them ineffective. A study in Greece analyzing a renal transplant cohort within the first three post-transplant years found lower Mg^2+^ levels in patients receiving supplements compared to those without supplementation [5]. Our study design excluded patients with chronic gastrointestinal losses to minimize confounding. The low bioavailability of some formulations along with poor adherence or unmeasured gastrointestinal losses could contribute to the limited response observed. However, our data suggest that Mg^2+^ supplementation may be insufficient and warrant further evaluation.

Another significant association with hypomagnesemia is DM. Hypomagnesemia alters the activity of pancreatic β-cells and modifies insulin secretion, while also promoting insulin resistance through the modulation of insulin receptor autophosphorylation [20,28]. Some studies consider hypomagnesemia a predictor of post-transplant DM [29,30].

Finally, considering that hypomagnesemia is the most frequent electrolytic disturbance and it is associated with multiple adverse outcomes, such as an increased risk of DM, hypertension, CVD [5,31,32], inflammation, immune regulation [33,34], and fatigue, among others, one of our goals should be to diagnose and prevent it whenever possible. This could translate into improved quality of life, morbidity, and mortality for our patients, which warrants the design of clinical studies.

The main limitation of the present study was that it was a retrospective, cross-sectional observational study of clinical practice. These studies are subject to multiple biases, particularly selection bias, making it essential for the sample to be representative, which is the case in our study. The primary limitation is the inability to establish a clear temporal sequence between the dependent variable and the independent variables or covariates, precluding causal inference. One of its strengths, however, is that such studies are useful for measuring prevalence.

Although our logistic regression model showed good discrimination (AUC = 0.77), its clinical utility remains to be determined. External validation is required but it may aid in identifying high-risk patients who could benefit from targeted monitoring or early interventions.

## 4. Materials and Methods

**Figure 4:**
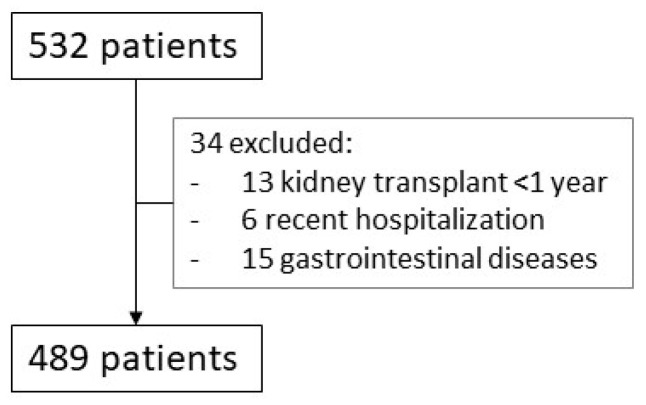
Screening process. All patients seen in the clinics between February and April 2023 were initially included (N = 532). After removing those who met the exclusion criteria, we analyzed 489 patients.

This was a retrospective, cross-sectional study based on data from all KTR attending routine check-ups at our outpatient clinics between February and April 2023. Cross-sectional studies analyze data from a population at a specific point in time, assessing the prevalence of an outcome and identifying associations between variables without establishing causality. Patients who had been hospitalized in the previous month or had underlying gastrointestinal conditions causing chronic diarrhea (favoring hypomagnesemia) or had undergone kidney transplantation less than one-year prior were excluded. A total of 523 patients were initially considered; 34 met the exclusion criteria, leaving 489 included in the final analysis (Figure 4).

Standard demographic, clinical, and laboratory data (including medication details) were recorded. Patients who were on Mg^2+^ supplements received oral Magnogene^®^ (by Uriach Laboratories), which contains 125.06 mg of magnesium hydroxide, 7.17 mg of magnesium bromide, and 0.34 mg of magnesium fluoride. All data were obtained in anonymized form from patients’ electronic medical records. No specific blood tests were ordered, as serum Mg^2+^ has been routinely included in follow-up laboratory panels for several years. To assess adherence to treatment, we reviewed the centers’ pharmacy fill database. All medications were collected. In ten individuals, there was a delay in collecting one of the monthly supplies, but it was less than 20 days. This database, part of the Madrid Community system [Módulo Único de Prescripción (MUP)], provides prescription details for all individuals residing in the region. Patient consent was waived due the retrospective and anonymized nature of the study. The study received approval from our center’s Ethics Committee on 6 March 2023.

Serum levels of ions (sodium, potassium, and chloride) were measured by indirect potentiometry on an AU 5800 analyzer (Beckman Coulter, Brea, CA, USA). Calcium, phosphorus, Mg^2+^, and creatinine were also measured using the AU 5800 analyzer. Calcium concentration was determined using the Arsenazo III colorimetric method, phosphorus using the phosphomolybdate method, Mg^2+^ using the Xylidyl Blue method, and creatinine using the standardized alkaline picrate methodIFCC-IDMS.

Parathyroid hormone was quantified by electrochemiluminescence immunoassay (ECLIA) on a Cobas e-411 analyzer (Roche Diagnostics, Mannheim, Germany). 25- OH vitamin D levels were determined using a chemiluminescent microparticle immunoassay (CMIA) on an Alinity i (Abbot, Abbot Park, IL, USA). Tacrolimus concentrations were measured using a homogeneous particle-enhanced turbidimetric immunoassay (QMS) on an Indiko analizer (ThermoFisher, Waltham, MA, USA).

### Statistical Analysis

Variables were treated as continuous or categorical according to their nature: variables with numerical values that can be measured on a scale (e.g., serum creatinine, eGFR…) were considered continuous. In contrast, variables with non-numerical values or binary responses (e.g., presence of hypomagnesemia, DM status, treatment with SGLT2i…) were considered categorical. Categorical variables are presented as absolute and relative frequencies. Continuous variables are described as mean and standard deviation or median and interquartile range (IQR), depending on their distribution. Normality of the variables was assessed using the Kolmogorov–Smirnov test. The chi-squared test or Fisher’s exact test was used to compare categorical variables. For continuous variables, we used the parametric Student’s *t*-test and non-parametric tests (Wilcoxon or Friedman) for normal/non-normal distributions.

To identify factors potentially associated with hypomagnesemia (serum Mg^2+^ levels ≤ 1.7 mg/dL), a univariate analysis was conducted, including demographic and analytical variables (sex, age, time post-transplant, DM status). Estimated glomerular filtration rate (eGFR) was calculated using the CKD-EPI equation [35]. Non parametric variables were stratified according the P75 value (serum calcium and phosphate). We also evaluated the use of different medications (immunosuppressants, diuretics, renin–angiotensin–aldosterone system inhibitors, cholecalciferol, calcitriol, paricalcitol, cinacalcet, denosumab, SGLT2i, PPIs, and oral calcium/magnesium supplements).

A logistic regression model was adjusted by backward stepwise regression based on maximum likelihood estimators. Variables with a *p* < 0.15 in the univariate analysis, as well as those considered clinically relevant based on previous studies, were included. After excluding collinearity, the optimal subset of variables was selected through backward elimination. Parameters were estimated with their corresponding 95% confidence interval (CI). To normalize residuals, serum calcium, phosphate PTH and 25(OH) vitamin D levels were stratified according to their 75th percentile (P75) values.

Odds ratios and their significance were calculated for each variable according to criteria for entry (*p* ≤ 0.05) and removal (*p* ≥ 0.10). Possible interactions were assessed by introducing multiplicative terms. The discriminative ability of the logistic models was determined through the area under the receiver operating characteristic curve (AUROC) and 95% CI. The Hosmer–Lemeshow test was used to assess goodness-of-fit. Model selection was based on that showing the highest discriminative power, good calibration, viable capacity and fulfilling the principle of parsimony (explaining the maximum variability outcome variable with the smallest number of variables included).

All statistical tests were performed on an intention-to-treat basis using the package SPSS version 25 (SPAA Inc., Chicago, IL, USA).

We used the STROBE cross-sectional checklist when writing our report [36].

## 5. Conclusions

In conclusion, hypomagnesemia is a common electrolyte disturbance in KTR, warranting routine monitoring of serum Mg^2+^ levels in this patients. Risk factors include tacrolimus, as well as cinacalcet and thiazides, which should be carefully considered in these patients. Oral Mg^2+^ supplements appear insufficient, highlighting the need for further research into alternative or additional managing strategies. Although our findings suggest a potential protective role of SGLT2i that could be beneficial in cases of severe hypomagnesemia, and given their additional renal and cardiovascular benefits, these observations require confirmation in prospective studies.

## Figures and Tables

**Table 1:** Demographic and laboratory data of KTR with low vs. normal serum Mg^2+^ levels.

Variable	Serum Magnesium Level		*p*
	Low (≤1.7 mg/dL) (N = 248)	Normal (≥1.8 mg/dL) (N = 241)	
Recipient age, mean (SD)	66.4 (14.1)	62.9 (13.2)	0.131
Recipient gender (male) N (%)	160 (49.8%)	161 (50%)	0.594
Diabetes mellitus N (%)	123 (49.4%)	126 (50.6%)	0.553
Months post-transplantation, median (IQR)	119.3 (46–99)	113 (60–172)	0.618
eFGR, median (IQR)	45.7 (34.0–63.2)	43.5 (31.8–57.5)	0.497
Serum magnesium (mg/dL), mean (SD)	1.56 (0.14)	1.96 (0.17)	<0.001
Serum calcium (mg/dL), median (IQR)	9.7 (9.4–10.0)	9.8 (9.4–10.1)	0.186
Serum phosphorus, median (IQR)	3.3 (3.0–3.7)	3.5 (3.0–3.9	0.002
25 OH vitamin D (ng/mL), median (IQR) (N = 465)	25 (18–33)	25 (18–33)	0.958
PTH (pg/mL), median (IQR) (N = 469)	98.7 (67.3–140.3)	100 (71.4–141.0)	0.818
Magnesium fractional excretion (%) (N = 443)	7.23 (4.80–9.99)	7.18 (5.05–10.15)	0.722
Urinary magnesium (mg/day), median (IQR) (N = 443)	630 (424–937)	768 (560–1039)	0.003
Urinary magnesium/creatinine ratio (mg/g) (N = 443)	56.9 (37.2–75.0)	66.5 (47.3–89.3)	<0.001

Comparison of clinical, demographic and laboratory data between patients with hypomagnesemia and those with normal Mg^2+^ levels. Continuous variables are shown as mean (SD) or median (IQR) and categorical variables as N (%). PTH, parathyroid hormone.

**Table 2:** Treatment of KTR with low vs. normal serum Mg^2+^ levels.

Variable	Serum Magnesium Level		*p*
	Low (≤1.7 mg/dL) (N = 248)	Normal (≥1.8 mg/dL) (N = 241)	
Tacrolimus treatment N (%)	217 (54.4%)	182 (45.6%)	0.001
Tacrolimus levels (ng/mL)	8.22 (2.76)	7.68 (2.01)	0.030
Cyclosporine treatment N (%)	16 (42.1%)	21 (56.8%)	0.344
Cyclosporine levels (ng/mL)	97.8 (29.7)	94.2 (26.3)	0.698
Mycophenolate treatment N (%)	190 (52.6)	171 (47.4)	0.155
Prednisone treatment N (%)	134 (50.6)	131 (49.4)	0.943
Azathioprine treatment N (%)	9 (60%)	6 (40%)	0.465
mTOR inhibitors treatment N (%)	52 (39.4%)	80 (60.6%)	0.002
Loop diuretics, treatment N (%)	22 (46.8%)	25 (53.2%)	0.573
Thiazide diuretics treatment N (%)	44 (62.9%)	26 (37.1%)	0.028
Cholecalciferol treatment	152 (51.7%)	142 (48.3%)	0.593
Calcitriol treatment N (%)	3 (30.3%)	7 (70%)	0.186
Paricalcitol treatment N (%)	25 (53.2%)	22 (46.8%)	0.721
Cinacalcet treatment N (%)	49 (63.6%)	28 (36.4%)	0.013
Proton pump inhibitors treatment N (%)	173 (50.7%)	168 (49.3%)	0.991
Denosumab treatment N (%)	12 (66.7%)	6 (33.3)	0.168
ACEI treatment N (%)	83 (56.1%)	65 (43.9%)	0.118
ARA2 treatment N (%)	127 (51%)	122 (49.0%)	0.897
MRA treatment N (%)	30 (45.5%)	36 (54.5)	0.358
iSGLT2 treatment N (%)	26 (26.0%)	94 (74.0%)	<0.001
Oral Mg^2+^ supplement	110 (75.9%)	35 (24.1%)	<0.001

Comparison of treatment between patients with hypomagnesemia and those with normal Mg^2+^ levels. Continuous variables are shown as mean (SD) or median (IQR) and categorical variables as n (%). PPi, proton pump inhibitors; ACEI, angiotensin-converting enzyme inhibitor; ARB, angiotensin receptor blocker; MRA, mineralocorticoid receptor antagonists; iSGLT2, sodium-glucose cotransporter-2 inhibitors.

**Table 3:** Logistic regression for hipoMg^2+^ ≤ 1.7 mg/dL.

Variable	OR (PIC)	*p*
Age	0.97 (0.96–0.99)	0.001
Months post-transplantation	1.005 (1.003–1.008)	<0.001
Diabetes mellitus No Si	1 1.94 (1.22–3.08)	0.005
iSGLT2 treatment No Yes	6.18 (3.64–10.5) 1	<0.001
Tacrolimus treatment No Yes	1 2.91 (1.62–5.22)	<0.001
Thiazide treatment No Yes	1 2.23 (1.21–4.08)	0.010
Cinacalcet treatment No Yes	1 2.31 (1.29–4.13)	0.005
IMTOR treatment No Yes	1.61 (1.02–2.62) 1	0.042
Serum phosphate * <3.7 mg/dL ≥3.7 mg/dL (p75)	1.99 (1.29–3.05) 1	0.002
Serum calcium * ≤10 mg/dL >10 mg/dL (p75)	1.75 (1.11–2.78) 1	0.017

Adjusted by sex, estimated filtration rate, use of cyclosporine and proton pump inhibitor use. * Calcium and phosphate values were selected because they correspond to the 75th percentile of our sample.

## Data Availability

The data that support the findings of this study are available from the corresponding author upon reasonable request. A private link to access the dataset during the peer review process is available via Figshare.

## Abbreviations

The following abbreviations are used in this manuscript:

ACEI	Angiotensin-converting enzyme inhibitor
ARB	Angiotensin receptor blocker
ATP	Adenosine triphosphate
CAV	Cardiac allograft vasculopathy
CVD	Cardiovascular disease
DCT	Distal convoluted tubule
DM	Diabetes mellitus
EGF	Epidermal growth factor
eGFR	Estimated glomerular filtration rate
HT	Heart transplant
KTR	Kidney transplant recipients
Mg^2+^	Magnesium
MRA	Mineralocorticoid receptor antagonists
mTORi	Mammalian target of rapamycin inhibitors
PPIs	Proton pump inhibitors
PTH	Parathyroid hormone
ROS	Reactive oxygen species
SGLT2i	Sodium-glucose cotransporter-2 inhibitors
TRPM6	Transient Receptor Potential Melastatin 6
TRPM7	Transient Receptor Potential cation channel subfamily M member 7
